# Reimplantation of an Anomalous Coronary Artery Arising from the Pulmonary Artery

**DOI:** 10.1155/2009/835459

**Published:** 2009-03-25

**Authors:** Andrea Quarti, Alessandro D'Alfonso, Massimo Colaneri, Alessandra Baldinelli, Maria Grazia Bettuzzi, Marco Pozzi

**Affiliations:** Department of Congenital and Paediatric Cardiac Surgery and Cardiology, Ospedali Riuniti, Presidio G.M. Lancisi, 60126 Ancona, Italy

## Abstract

A case of anomalous origin of the left coronary artery from the pulmonary artery in a patient with the origin of the coronary opposite to the aorta is reported. Between many surgical options we conclude to reestablish a double coronary system reconnecting the coronary through a conduit created with a pulmonary wall baffle and an autologous pericardial patch.

## 1. Introduction

Anomalous origin of the left coronary artery arising from the pulmonary artery (ALCAPA) is a rare congenital anomaly. The anatomic characteristics of the relation between the ALCAPA and the aorta are the major issue for the choice of the repairing technique.

## 2. Case Report

A 20-day-old boy was referred to our institution with signs of heart failure and low ejection fraction associated with mild-to-moderate mitral regurgitation (MR). At a physical examination, a systolic murmur was noted at the left sternal border. The chest X-ray showed an enlargement of the heart with pulmonary plethora.

An echocardiographic examination showed a dilated right coronary artery with retrograde flow through the left coronary artery into the main pulmonary artery. A diagnosis of ALCAPA was made. At examination, a mild-to-moderate MR was also founded, related to ventricular dysfunction (EF 25%) with a central jet, thetering of the mitral leaflets and fibrosis of the anterior papillary muscles. The patient didn’t require preoperative inotrops and was under intravenous infusion of furosemide.

The operation was performed through a median sternotomy on cardiopulmonary bypass using bicaval cannulation and moderate hypotermia. Myocardial protection was achieved by antegrade cardioplegia through the aortic root after cross-clamping the aorta and snaring of both the branches of the pulmonary artery to ensure efficient delivery of cardioplegia. We didn’t attempt to repair the mitral valve at the time of coronary relocation.

**Figure 1:**
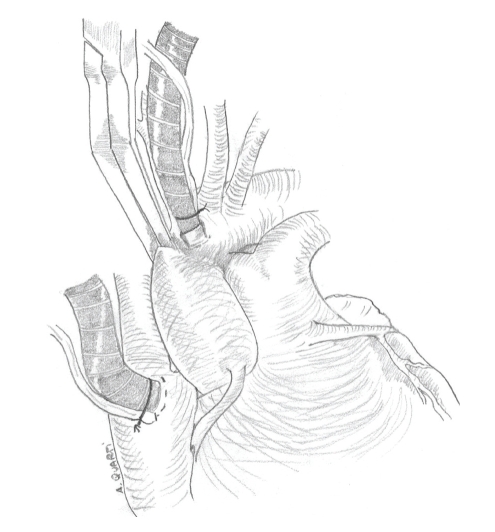
Coronary anatomy.

**Figure 2:**
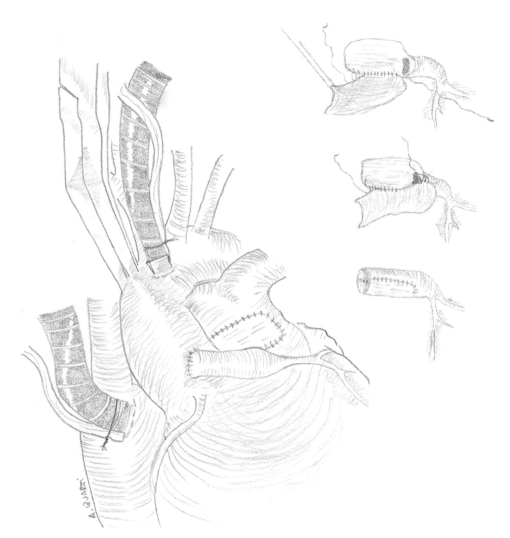
Creation and relocation of the conduit.

Over the last 14 years we have used the reimplantation technique in all the cases. In our patient the left coronary artery arised opposite to the aorta from the nonfacing sinus (Figure 1) precluding the direct relocation. We explanted the coronary artery with a pulmonary artery wall baffle and reconstructed a conduit using the baffle and an autologous pericardial patch (Figure 2). The conduit obtained was then anastomosed on the aorta, and the pulmonary artery was finally reconstructed with autologous pericardial patch (Figure 2). The patient was easily weaned from cardiopulmonary bypass on a moderate amount of inotropic support. The patient was extubated on the third postoperative day and the daily echocardiographic examination depicted a slight but progressive improvement in the ejection fraction. One week after the procedure the EF was 50% while the MR was mild.

The patient was discharged from the hospital 13 days after the operation. At the last follow-up (6 months) consisting of an echocardiography, the left ventricle was well functioning while the MR is still mild despite the reduction of the ventricular volume.

## 3. Comment

Anomalous origin of the left coronary artery from the pulmonary artery is a rare condition leading to high mortality in the first year of life [1]. The most popular surgical correction is the direct relocation, firstly described by Neches et al. [2]. The major issue in applying this technique is the coronary anatomy and in particular the distance between the anomalous origin of the left coronary and the aorta. Takeuchi et al. [3] described a technique which creates a tunnel inside the pulmonary artery between the coronary ostia and a surgically created aorto-pulmonary window. Potential complications of this technique include: supra-valvular pulmonary stenosis, coronary-PA fistula, aortic regurgitation, and reoperations or catheter interventions which are necessary in almost 30% of patients after the procedure [4].

Isolated ALCAPA ligation has also been described, but this is the only technique leading to a reduced long-term survival. This technique also precludes the normalization of LV ejection fraction or volume [5, 6]. No differences in long-term survival have been demonstrated between the other techniques leading to a two-coronary system [5–7].

Our policy is to reimplant directly the ALCAPA on the aorta, but in our patient the distance between the coronary origin in the nonfacing sinus of the pulmonary artery and the aorta precluded a direct reimplantation. In order to avoid the complications described with the Takeuchi technique we constructed an external conduit with the use of autologous pericardium and reconnected the coronary artery through this extension conduit to the aorta. The conduit hence obtained was then relocated on the aorta (Figure 2).

Mitral valve repair was not considered because in our previous experiences [8] we noticed the improvement in mitral valve function after the operation probably due to the decrease of annular size with the reduction in left ventricular dimensions and the improvement in papillary muscles perfusion.

This technique is a very simple and fast solution to those anatomical conditions precluding the direct relocation of the coronary artery to the aorta. However, although the early follow-up is very encouraging; the follow-up is too short to exclude the possibility of medium- to long-term complication.
